# Metastatic basal cell carcinoma with amplification of PD-L1: exceptional response to anti-PD1 therapy

**DOI:** 10.1038/npjgenmed.2016.37

**Published:** 2016-10-19

**Authors:** Sadakatsu Ikeda, Aaron M Goodman, Philip R Cohen, Taylor J Jensen, Christopher K Ellison, Garrett Frampton, Vincent Miller, Sandip P Patel, Razelle Kurzrock

**Affiliations:** 1 Department of Medicine, Division of Hematology/Oncology, University of California, La Jolla, CA, USA; 2 Center for Personalized Cancer Therapy, University of California San Diego, Moores Cancer Center, La Jolla, CA, USA; 3 Cancer Center, Tokyo Medical and Dental University, Tokyo, Japan; 4 Department of Dermatology, University of California San Diego, La Jolla, CA, USA; 5 Sequenom Laboratories, La Jolla, CA, USA; 6 Foundation Medicine, Cambridge, MA, USA

## Abstract

Metastatic basal cell carcinomas are rare malignancies harbouring Hedgehog pathway alterations targetable by SMO antagonists (vismodegib/sonidegib). We describe, for the first time, the molecular genetics and response of a patient with Hedgehog inhibitor-resistant metastatic basal cell carcinoma who achieved rapid tumour regression (ongoing near complete remission at 4 months) with nivolumab (anti-PD1 antibody). He had multiple hallmarks of anti-PD1 responsiveness including high mutational burden (>50 mutations per megabase; 19 functional alterations in tissue next-generation sequencing (NGS; 315 genes)) as well as *PDL1/PDL2/JAK2* amplification (as determined by both tissue NGS and by analysis of plasma-derived cell-free DNA). The latter was performed using technology originally developed for the genome-wide detection of sub-chromosomal copy-number alterations (CNAs) in noninvasive prenatal testing and showed numerous CNAs including amplification of the 9p24.3-9p22.2 region containing *PD-L1*, *PD-L2* and *JAK2*. Of interest, *PD-L1*, *PD-L2* and *JAK2* amplification is a characteristic of Hodgkin lymphoma, which is exquisitely sensitive to nivolumab. In conclusion, selected SMO antagonist-resistant metastatic basal cell carcinomas may respond to nivolumab based on underlying molecular genetic mechanisms that include *PD-L1* amplification and high tumour mutational burden.

## Introduction

Basal cell carcinoma is the most common malignancy, with an estimated 2.8 million Americans diagnosed annually.^1^ These cutaneous tumours are usually indolent and cured by local resection. However, basal cell carcinomas can be locally destructive; rarely, they metastasise to distant organs. The incidence of metastatic basal cell carcinoma is reported to be between 0.0028 and 0.55%, depending on the case series. Metastatic lesions typically have pathologic features similar to those in the primary tumour: vascular or perineural invasion can be associated with metastasis. With traditional combined modality therapy utilising surgery, chemotherapy and radiation, the prognosis of metastatic basal cell carcinoma is poor.

Molecular and genomic studies of basal cell naevus syndrome (BCNS), a rare inherited form of basal cell carcinoma, lead to the discovery of the *PTCH1* mutation on chromosome 9.^2^ PTCH1 is a receptor for Sonic Hedgehog, and negatively regulates the Hedgehog pathway by inhibiting smoothened (SMO), a Frizzled class receptor. PTCH1 is a tumour suppressor gene, and the loss of PTCH1 is sufficient to induce tumorigenesis *in vivo*.^3^ Overexpression of SMO is sufficient to cause a basal cell carcinoma-like lesion in a mouse model.^4^ Close to 100% of basal cell carcinomas harbour mutations in the Hedgehog pathway with ~90% of tumours having mutations in *PTCH1*.^5^ On the basis of this discovery, molecularly targeted therapy aimed at the Hedgehog pathway was developed. Vismodegib and sonidegib are two SMO antagonists approved by the Food and Drug Administration (FDA) for locally advanced or metastatic basal cell carcinoma. Pivotal studies demonstrated objective response rates in approximately 30–45% of patients with a median duration of response over six months.^6,7^

Although Hedgehog pathway inhibitors may result in an initial response, basal cell carcinoma generally develops resistance, sometimes because of secondary mutations in the *SMO* gene.^8,9^ In the current study, we performed hybrid-capture based next-generation sequencing (NGS) of tumour tissue as well as analysis of circulating cell-free DNA (cfDNA) for genome-wide copy-number changes in a patient with vismodegib- and sonidegib-resistant metastatic basal cell carcinoma whose tumour harboured a *PTCH1* mutation.^10^ Unexpectedly, we discovered multiple mutations and copy-number alterations (CNAs), with the former considered a marker for response to immunotherapy in solid tumours.^11,12^ In addition, there was amplification of programmed death ligand 1 (*PD-L1,* also called *CD274*), programmed death ligand 2 (*PD-L2*), and Janus kinase 2 (*JAK2*), a hallmark of the 9p24.1 amplicon seen in nodular sclerosing Hodgkin lymphoma and primary mediastinal large B-cell lymphoma.^13^ It has been hypothesised that chromosome 9p24.1 (the locus for PD-L1, PD-L2, and JAK2) amplification allows the neoplastic Reed–Sternberg cells to evade immune system recognition through up-regulation of PD-L1 and/or PD-L2. This was proven true as single agent nivolumab demonstrated a remarkable overall response rate of 87% in patients with relapsed/refractory Hodgkin lymphoma.^14^ Our patient with metastatic basal cell carcinoma, with multiple genomic alterations and *PD-L1/PD/L2/JAK2* amplification, also had an exceptional response to single agent nivolumab.

## Case report

A 58-year-old man with a history of frequent visits to sun tanning salons developed basal cell carcinoma located on his face that was treated by resection 10 years ago. Six years later, he developed a recurrent basal cell carcinoma on the left posterior shoulder that required multiple surgical resections and post-operative radiation due to involved margins. Two years ago, he presented with new onset back pain and was found to have metastatic disease involving the axial skeleton, lungs and liver. An L4 vertebral bone biopsy demonstrated metastatic carcinoma with basaloid features. The tumour was positive by immunohistochemistry (IHC) for p63, keratin 5/6, and negative for keratin 7, TTF-1 and keratin 20. This immunophenotype was identical to the immunophenotype of the prior basal cell carcinoma involving his shoulder. Pathologic tissue from metastases reviewed at MD Anderson Cancer Center and at UC San Diego confirmed the diagnosis of metastatic basal cell carcinoma. Shown in Table 1 are the results of hybrid-capture based NGS^15^ performed by Foundation Medicine (Supplementary Tables 1 and 2 http://www.foundationone.com/). This is a commercially available, NGS-based panel (clinical-grade) comprising 315 genes for the purpose of looking for clinically actionable somatic genomic variation. This panel initially demonstrated ten functional genomic alterations including *PTCH1* Q1366*, W197* and *CDKN2A* P81L, with an overall tumour mutational burden (TMB) of 79 base substitution and indel mutations per megabase (Mb) of coding genome. The patient was managed in accordance with UCSD IRB guidelines and signed consent for investigational treatments, diagnostics, and follow up.

He was started on vismodegib 1.5 years ago. Two months later he developed a seizure. Magnetic resonance imaging (MRI) of his brain showed a 2.7×1.7 cm right frontal lobe peripherally enhancing mass associated with a small amount of adjacent vasogenic oedema. He underwent stereotactic radiosurgery to the right frontal lobe lesion. This was followed by four cycles of cisplatin and paclitaxel. PET/CT scan two months later demonstrated a partial response to therapy with resolution of most of the lesions in his liver and lungs. However, he did not tolerate cisplatin and paclitaxel; he developed symptomatic anaemia, fatigue, depression, severe loss of appetite and weight loss. In addition, PET/CT scan demonstrated progressive disease in his skeleton and liver. Chemotherapy was discontinued.

Two months later, he was started on a clinical trial with sonidegib combined with buparlisib, a pan-class I PIK3 inhibitor. Six weeks later, CT scans demonstrated progressive disease in his liver, and therapy was switched to weekly paclitaxel and vismodegib. He remained on paclitaxel and vismodegib for two months; treatment was complicated by pneumonia requiring hospital admission. Therapy was discontinued due to poor tolerance and lack of response.

**Figure 1:**
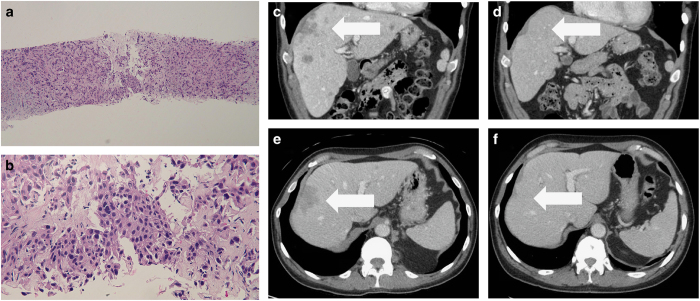
Histology of metastatic basal cell Carcinoma sample—medium (**a**) and high (**b**) magnification views of liver biopsy and CT scans demonstrating near complete response to PD-1 blockade (**e**, **f**). The metastatic tumour extensively infiltrates the liver; several tumour cells have large pleomorphic nuclei (**a**). Aggregates of the basaloid tumour cells are also present (**b**). (Hematoxylin and eosin: ; ×10, **a**; ×40, **b**). Baseline CT of liver lesions before treatment (**c**, **e**). Near complete resolution of the liver lesion after nivolumab for four months (**d**, **f**). The white arrows demonstrate metastatic basal cell carcinoma in the liver prior to (**c**, **e**) and following (**d**, **f**) 4 months of treatment with nivolumab.

Repeat NGS, shown in Table 1, was performed on a liver biopsy specimen (Figure 1a,b). Sequencing demonstrated multiple new genomic alterations including amplification of *PD-L1*, *PD-L2* and *JAK2,* in addition to 16 other functional mutations and a TMB of 103 mutations per Mb. The result was presented in the multidisciplinary Molecular Tumor Board, and anti-PD1 therapy was discussed.^16^

**Figure 2:**
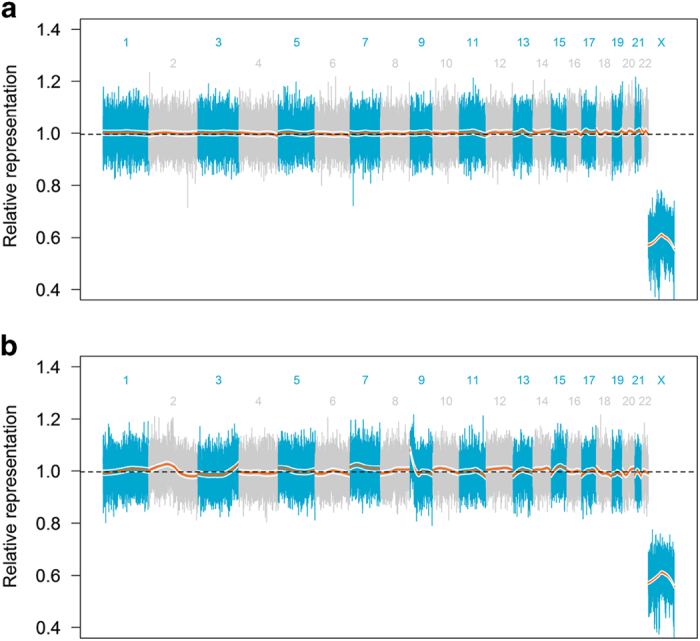
Genome-wide sequencing of cfDNA using non-invasive prenatal testing (NIPT) technology. Sequencing reads were assigned to 50 kilobase (kb) non-overlapping segments of the human reference genome. The normalised read counts from each segment of the genome are shown with alternating colours delineating chromosomes and the number of each chromosome shown. A LOESS regression was performed for each chromosome (orange lines) with 95% confidence intervals shown (white lines). Deviations above and below the median value (dashed black line) indicate amplifications and deletions, respectively. (**a**) Genome-wide profiles from a patient with Erdheim–Chester disease that was sequenced as a comparator. The lack of deviations of genomic segments above or below the median representation is shown to depict a sample without copy-number alterations. (**b**) Genome-wide profile of this patient with basal cell carcinoma. In addition to amplification of 9p24.3-9p22.2, numerous copy-number alterations are shown throughout the genome as indicated by the deviation of these regions away from the median value.

CfDNA was also isolated from plasma and analysed for genome-wide CNAs using Sequenom Laboratories technology originally developed for the genome-wide detection of sub-chromosomal CNAs in noninvasive prenatal testing (Figure 2).^10^ In addition to the amplification of the region containing *PD-L1*, *PD-L2* and *JAK2* (amplification defect spanning 9p24.3-9p22.2), this analysis detected numerous significant CNAs across the genome.

He was started on nivolumab 240 mg intravenously every 2 weeks. Within 2 weeks, his fatigue improved. Two months after starting nivolumab, CT scans demonstrated a marked decrease in size of the liver lesions. Four months after starting nivolumab, CT scans demonstrated near complete resolution in the hepatic lesions (Figure 1c–f), and his mood and appetite improved.

## Discussion

Our patient had *PTCH1*-mutated metastatic basal carcinoma that had progressed following vismodegib, sonidegib and cytotoxic chemotherapy, leaving him with limited options for further therapy. A case report has shown incidental regression of an advanced basal carcinoma in a patient with metastatic melanoma being treated with ipilimumab, a cytotoxic T-lymphocyte associated protein-4 (CTLA-4) antagonist, implying that these tumours can be responsive to checkpoint inhibition.^17^ Recently, Winkler *et al.*^18^ reported on a patient with metastatic basal cell carcinoma whose best response to treatment with pembrolizumab was stable disease for a few months. PD-L1 expression by IHC was found to be low in the patient’s pulmonary metastases. NGS and tumour mutational burden were not reported. Our report differs in several key aspects. Our patient had a marked and durable response to anti-PD1 therapy. In addition, we present NGS data that demonstrated amplification of 9p24.1 and high TMB providing a biologic rationale as to why basal cell carcinoma might respond to PD-1 blockade. Furthermore, we present analysis of circulating cfDNA demonstrating diffuse genome-wide copy-number changes including the amplification of chromosome 9p24.1.

Our patient’s rapid response to PD-1 blockade with nivolumab suggests immune evasion as a key mechanism of tumour growth and maintenance. Supporting this biological hypothesis, NGS revealed amplification of *PD-L1, PD-L2,* and *JAK2,* all located in close proximity on chromosome 9p24.1. PD-L1 and PD-L2, along with programmed cell death protein-1 (PD-1), are key components of the programmed cell death immune checkpoint. Tumour cells take advantage of this checkpoint by expressing PD-L1 and/or PD-L2, which bind to PD-1 on CD8+ cytotoxic T cells, resulting in T-cell exhaustion and apoptosis.^19^ The end result is protection of tumour cells from immune-mediated destruction. Immune checkpoint blockade with monoclonal antibodies works by suppressing these key regulatory proteins involved in immune down regulation, with the goal of stimulating T-cell-dependent cellular cytotoxicity against tumour cells. This has been successfully demonstrated in melanoma and some non-small cell lung cancers, which have been shown to have impressive and durable responses to PD-1 blockade with monoclonal antibodies.^20,21^

Amplification of 9p24.1 loci has been reported in the majority of patients with classical Hodgkin lymphoma.^13^ This structural anomaly increases gene dosage of PD-L1, defining the PD-1 pathway as a rational therapeutic target. This was proven true in a pivotal study evaluating nivolumab in a group of 23 relapsed/refractory Hodgkin lymphoma patients;^14^ 78% of these patients had relapsed after autologous transplantation, and 78% had relapsed after treatment with brentuximab vedotin. The overall response rate to nivolumab was 87%. Amplification of 9p24.1 also results in *JAK2* amplification. JAK2 is able to further increase PD-L1 production by augmenting the signal transducer and activator of transcription (STAT) protein, resulting in increased PD-L1 transcription.^13^ Therefore, both increased copy number of PD-L1 and increased JAK2 signalling result in up regulation of PD-L1 transcription in Reed–Sternberg cells.

In addition to Hodgkin lymphoma, amplification of chromosome 9p24.1 has been found in 19% of squamous cell carcinomas of the oral cavity, 29% of triple-negative breast cancer, 5% of glioblastoma and 3% of colon cancer.^22,23^ Recently, it has been shown that ~20% of patients with triple-negative breast cancer respond to PD-1 blockade with pembrolizumab.^24^ Chromosome 9p24.1 amplification appears to be a recurrent event across several tumour types and further studies will need to be performed to assess its value as a biomarker for response to PD-1 blockade.^25^

CD8+ T-cell-mediated destruction of tumour cells relies upon presentation of tumour antigen bound to major histocompatibility class-1 (MHC-1) on the surface of antigen-presenting cells to CD+8 T-cells. Antigens must be sufficiently distinct from self to elicit an immune response. Tumour antigens that are not previously recognised by the immune system have been termed neoantigens. Higher neoantigen burden, which may be largely predicted by TMB, has been associated with higher objective response rates and progression-free survival in patients treated with PD-1 blockade.^11,12^ Melanoma, non-small cell lung cancers, and renal cell carcinoma, all of which are highly responsive to PD-1 blockade, have been shown to have a high degree of mutational burden from whole genome and exome sequencing studies.^26^

Our experience suggests that the median number of alterations in an NGS panel of ~200 to 300 genes used in metastatic disease is about 4.^27^ Although this is a limited gene panel, the sequencing of ~1.5 Mb across the subset of cancer related genes allowed detection of 19 functional alterations. Compared with most tumours, basal cell carcinoma has a very high overall TMB with a median of 50 mutations per megabase (Table 2).^28^ Our patient had a level of >100 total mutations per Mb of coding genome, which corresponds to >1000 mutations in the whole exome and represents a very high TMB, even for basal cell carcinoma.

In addition to single-nucleotide variants, tumours often exhibit widespread CNAs related to genome instability. We sequenced the cfDNA from the plasma of this patient to 0.2× coverage and detected CNAs using methods developed for noninvasive prenatal testing.^29^ Numerous significant CNAs were detected across the entire genome including clear amplification of the entire region surrounding these genes (9p24.3-9p22.2; Figure 2b). It is not known whether the CNAs found in the circulating cfDNA are the same that would be found in analysis of the liver metastases. Circulating cfDNA could represent tumour DNA from any of the patient’s tumours. Finding diffuse genome-wide CNAs and, specifically, CNAs in chromosome 9p24.1 further confirms amplification of chromosome 9p24.1 that was detected by NGS of the liver metastases. We hypothesise that the CNAs detected in cfDNA may be consistent with genomic instability and the high mutational burden in the tissue DNA derived from this patient. The technology used to detect CNAs in our patient’s circulating cell-free tumour DNA is identical to the method for noninvasive prenatal testing that has been used in over 500 000 pregnant women to detect fetal chromosome anomalies.^29^ Using this technology to detect tumour genomic alterations is a novel way to obtain valuable genomic information from a patient’s tumour with out necessitating a tissue biopsy.

Genomic profiling of our patient’s tumour allowed us to choose an effective treatment that would not have otherwise been considered in an individual with metastatic basal cell carcinoma. NGS technologies are already allowing oncologists to effectively customise treatment for their patients by matching therapies, such as tyrosine kinase inhibitors, with the appropriate mutation.^27,30,31^ In addition, certain types of NGS, such as hybrid-capture-based comprehensive sequencing of a broad panel of cancer genes, has the ability to identify other alterations that can predict a response to immunotherapy, including degree of mutational burden, errors in mismatch repair proteins, and PD-L1 amplification.^32^

In summary, we report an exceptional responder to anti-PD1 therapy in a patient with metastatic basal cell carcinoma whose tumour harboured co-amplification of *PD-L1*, *PD-L72*, and *JAK2* as well as multiple genomic alterations. There is no effective treatment option beyond the Hedgehog inhibitors in metastatic basal cell carcinoma. Therefore, there is an unmet need for new strategies beyond Hedgehog inhibitors. Our report suggests the merit of exploring biomarker-driven, immune-targeted therapy in metastatic basal cell carcinoma and in other tumours harbouring amplification of *PD-L1* and/or high tumour mutational burden.

## Figures and Tables

**Table 1:** Next-generation sequencing of biopsy specimens^a^

*Biopsy (timing)*	*Molecular alterations*
Soft tissue (pre-systemic chemotherapy)	***PTCH1***p.Q1366*, p.W197*
	*CDKN1A* p.R140Q
	*CDKN2A* p.P81L
	*CTNNA1* p.R383H
	*LRP1B* splice site c.9121-1G>A
	*NOTCH1* p.W287*
	*SLIT2* p.K325*
	*SMARCA4* p.Q1166*
	*TP53* p.P278S
	
Liver (post-systemic chemotherapy)	***CD274 (PD-L1)*** **amplification** ***JAK2 amplification*** ***PDCD1LG2 (PD-L2)*****amplification**
	*CDKN1A* p.R140Q *CDKN2A* p.P81L
	*CTNNA1* p.R383H
	*FLT1* p.E487K
	*LRP1B* splice site c.9121-1G>A, p.W2334* *MLL2* splice site c.4132-1G>A *NOTCH1* p.W287* *PDGFRA* p.E459K *PIK3R2* p.Q412* ***PTCH1***p.Q1366*, p.W197* *SLIT2* p.K325* *SMARCA4* p.Q1166* *TERT* promoter c.-139-138CC>TT
	*TP53* p.P278S

a Foundation Medicine.

Pertinent genomic alterations for the treatment of this patient are indicated in bold. Mutated gene are indicated in italic.

**Table 2:** Distribution of tumour mutational burden in 79 consecutive cases of basal cell carcinoma, as determined by next generation sequencing based comprehensive genomic profiling, in the course of routine clinical cancer care.^28^

*Tumour mutational burden (mutations per megabase)*	*Percentile*
215	Maximum
131	90th
79	75th
50	50th (median)
17	25th
5	10th
1	Minimum
58	Average

## References

[bib1] Gandhi, S. A. & Kampp, J. Skin cancer epidemiology, detection, and management. Med. Clin. North Am. 99, 1323–1335 (2015). 26476255 10.1016/j.mcna.2015.06.002

[bib2] Hahn, H. et al. Mutations of the human homolog of Drosophila patched in the nevoid basal cell carcinoma syndrome. Cell 85, 841–851 (1996). 8681379 10.1016/s0092-8674(00)81268-4

[bib3] Adolphe, C., Hetherington, R., Ellis, T. & Wainwright, B. Patched1 functions as a gatekeeper by promoting cell cycle progression. Cancer Res. 66, 2081–2088 (2006). 16489008 10.1158/0008-5472.CAN-05-2146

[bib4] Xie, J. et al. Activating Smoothened mutations in sporadic basal-cell carcinoma. Nature 391, 90–92 (1998). 9422511 10.1038/34201

[bib5] Epstein, E. H. Basal cell carcinomas: attack of the hedgehog. Nat. Rev. Cancer 8, 743–754 (2008). 18813320 10.1038/nrc2503 PMC4457317

[bib6] Sekulic, A. et al. Efficacy and safety of vismodegib in advanced basal-cell carcinoma. N. Engl. J. Med. 366, 2171–2179 (2012). 22670903 10.1056/NEJMoa1113713 PMC5278761

[bib7] Migden, M. R. et al. Treatment with two different doses of sonidegib in patients with locally advanced or metastatic basal cell carcinoma (BOLT): a multicentre, randomised, double-blind phase 2 trial. Lancet Oncol. 16, 716–728 (2015). 25981810 10.1016/S1470-2045(15)70100-2

[bib8] Sharpe, H. J. et al. Genomic analysis of smoothened inhibitor resistance in basal cell carcinoma. Cancer Cell 27, 327–341 (2015). 25759019 10.1016/j.ccell.2015.02.001 PMC5675004

[bib9] Atwood, S. X. et al. Smoothened variants explain the majority of drug resistance in basal cell carcinoma. Cancer Cell 27, 342–353 (2015). 25759020 10.1016/j.ccell.2015.02.002 PMC4357167

[bib10] Zhao, C. et al. Detection of fetal subchromosomal abnormalities by sequencing circulating cell-free DNA from maternal plasma. Clin. Chem. 61, 608–616 (2015). 25710461 10.1373/clinchem.2014.233312

[bib11] Rizvi, N. A. et al. Cancer immunology. Mutational landscape determines sensitivity to PD-1 blockade in non-small cell lung cancer. Science 348, 124–128 (2015). 25765070 10.1126/science.aaa1348 PMC4993154

[bib12] George T. J. et al. Tumor mutational burden as a potential biomarker for PD1/PD-L1 therapy in colorectal cancer [Abstract]. J. Clin. Oncol. 34 (2016).

[bib13] Green, M. R. et al. Integrative analysis reveals selective 9p24.1 amplification, increased PD-1 ligand expression, and further induction via JAK2 in nodular sclerosing Hodgkin lymphoma and primary mediastinal large B-cell lymphoma. Blood 116, 3268–3277 (2010). 20628145 10.1182/blood-2010-05-282780 PMC2995356

[bib14] Ansell, S. M. et al. PD-1 Blockade with Nivolumab in relapsed or refractory Hodgkin’s lymphoma. N. Engl. J. Med. 372, 311–319 (2015). 25482239 10.1056/NEJMoa1411087 PMC4348009

[bib15] Frampton, G. M. et al. Development and validation of a clinical cancer genomic profiling test based on massively parallel DNA sequencing. Nat. Biotechnol. 31, 1023–1031 (2013). 24142049 10.1038/nbt.2696 PMC5710001

[bib16] Schwaederle, M. et al. Molecular Tumor Board: The University of California San Diego Moores Cancer Center Experience. Oncologist 19, 631–636 (2014). 24797821 10.1634/theoncologist.2013-0405 PMC4041669

[bib17] Mohan, S. V., Kuo, K. Y. & Chang A. L. S. Incidental regression of an advanced basal cell carcinoma after ipilimumab exposure for metastatic melanoma. JAAD Case Rep. 2, 13–15 (2016). 27051815 10.1016/j.jdcr.2015.11.007 PMC4809440

[bib18] Winkler J. k. et al. Anti-PD-1 therapy in nonmelanoma skin cancer. Br. J. Dermatol (e-pub ahead of print 8 April 2016; doi:10.1111/bjd.14664).

[bib19] Dong, H. et al. Tumor-associated B7-H1 promotes T-cell apoptosis: a potential mechanism of immune evasion. Nat. Med. 8, 793–800 (2002). 12091876 10.1038/nm730

[bib20] Robert, C. et al. Nivolumab in previously untreated melanoma without BRAF mutation. N. Engl. J. Med. 372, 320–330 (2015). 25399552 10.1056/NEJMoa1412082

[bib21] Garon, E. B. et al. Pembrolizumab for the treatment of non-small-cell lung cancer. N. Engl. J. Med. 372, 2018–2028 (2015). 25891174 10.1056/NEJMoa1501824

[bib22] Straub, M. et al. CD274/PD-L1 gene amplification and PD-L1 protein expression are common events in squamous cell carcinoma of the oral cavity. Oncotarget 7, 12024–12034 (2016). 26918453 10.18632/oncotarget.7593 PMC4914266

[bib23] Barrett, M. T. et al. Genomic amplification of 9p24.1 targeting JAK2, PD-L1, and PD-L2 is enriched in high-risk triple negative breast cancer. Oncotarget 6, 26483–26493 (2015). 26317899 10.18632/oncotarget.4494 PMC4694916

[bib24] Nanda, R. et al. Pembrolizumab in patients with advanced triple-negative breast cancer: phase Ib KEYNOTE-012 study. J. Clin. Oncol. 34, 2460–2467 (2016). 27138582 10.1200/JCO.2015.64.8931 PMC6816000

[bib25] Patel, S. P. & Kurzrock, R. PD-L1 expression as a predictive biomarker in cancer immunotherapy. Mol. Cancer Ther. 14, 847–856 (2015). 25695955 10.1158/1535-7163.MCT-14-0983

[bib26] Alexandrov, L. B. et al. Signatures of mutational processes in human cancer. Nature 500, 415–421 (2013). 23945592 10.1038/nature12477 PMC3776390

[bib27] Schwaederle, M. et al. On the road to precision cancer medicine: analysis of genomic biomarker actionability in 439 patients. Mol. Cancer Ther. 14, 1488–1494 (2015). 25852059 10.1158/1535-7163.MCT-14-1061

[bib28] Frampton G. M. et al. Assessment of tumor mutation burden from >60,000 clinical cancer patients using comprehensive genomic profiling [Abstract]. J. Clin. Oncol. 34 (2016).

[bib29] Lefkowitz, R. B. et al. Clinical validation of a noninvasive prenatal test for genomewide detection of fetal copy number variants. Am. J. Obstet. Gynecol. 215, 227.e1–227.e16 (2016). 26899906 10.1016/j.ajog.2016.02.030

[bib30] Wheler, J., Lee, J. J. & Kurzrock, R. Unique molecular landscapes in cancer: implications for individualized, curated drug combinations. Cancer Res 74, 7181–7184 (2014). 25326492 10.1158/0008-5472.CAN-14-2329 PMC4292868

[bib31] Schwaederle, M. et al. Precision oncology: The UC San Diego Moores Cancer Center PREDICT Experience. Mol. Cancer Ther. 15, 743–752 (2016). 26873727 10.1158/1535-7163.MCT-15-0795

[bib32] Le, D. T. et al. PD-1 blockade in tumors with mismatch-repair deficiency. N. Engl. J. Med. 372, 2509–2520 (2015). 26028255 10.1056/NEJMoa1500596 PMC4481136

