# Supplementary material for: Metastatic basal cell carcinoma with amplification of PD-L1: exceptional response to anti-PD1 therapy
Source: NPJ Genom Med. 2016 Oct 19;1:16037–. doi: 10.1038/npjgenmed.2016.37 (PMC5142752; doi:10.1038/npjgenmed.2016.37)
Supplement: Supplementary Tables 1 and 2 [file npjgenmed201637-s1.doc]

**Supplemental table 1**: 315 cancer-related genes detected on the Foundation Medicine Panel.

| ABL1 | C11orf30 (EMSY) | DDR2 | FGFR4 | IL7R | MET | PIK3CA | SDHD | TSHR |
| --- | --- | --- | --- | --- | --- | --- | --- | --- |
| ABL2 | CARD11 | DICER1 | FH | INHBA | MITF | PIK3CB | SETD2 | U2AF1 |
| ACVR1B | CBFB | DNMT3A | FLCN | INPP4B | MLH1 | PIK3CG | SF3B1 | VEGFA |
| AKT1 | CBL | DOT1L | FLT1 | IRF2 | MPL | PIK3R1 | SLIT2 | VHL |
| AKT2 | CCND1 | EGFR | FLT3 | IRF4 | MRE11A | PIK3R2 | SMAD2 | WISP3 |
| AKT3 | CCND2 | EP300 | FLT4 | IRS2 | MSH2 | PLCG2 | SMAD3 | WT1 |
| ALK | CCND3 | EPHA3 | FOXL2 | JAK1 | MSH6 | PMS2 | SMAD4 | XPO1 |
| AMER1  (FAM123B) | CCNE1 | EPHA5 | FOXP1 | JAK2 | MTOR | POLD1 | SMARCA4 | ZBTB2 |
| APC | CD274 | EPHA7 | FRS2 | JAK3 | MUTYH | POLE | SMARCB1 | ZNF217 |
| AR | CD79A | EPHB1 | FUBP1 | JUN | MYC | PPP2R1A | SMO | ZNF703 |
| ARAF | CD79B | ERBB2 | GABRA6 | KAT6A (MYST3) | MYCL (MYCL1) | PRDM1 | SNCAIP |  |
| ARFRP1 | CDC73 | ERBB3 | GATA1 | KDM5A | MYCN | PREX2 | SOCS1 |  |
| ARID1A | CDH1 | ERBB4 | GATA2 | KDM5C | MYD88 | PRKAR1A | SOX10 |  |
| ARID1B | CDK12 | ERG | GATA3 | KDM6A | NF1 | PRKCI | SOX2 |  |
| ARID2 | CDK4 | ERRFI1 | GATA4 | KDR | NF2 | PRKDC | SOX9 |  |
| ASXL1 | CDK6 | ESR1 | GATA6 | KEAP1 | NFE2L2 | PRSS8 | SPEN |  |
| ATM | CDK8 | EZH2 | GID4  (C17orf39) | KEL | NFKBIA | PTCH1 | SPOP |  |
| ATR | CDKN1A | FAM46C | GLI1 | KIT | NKX2-1 | PTEN | SPTA1 |  |
| ATRX | CDKN1B | FANCA | GNA11 | KLHL6 | NOTCH1 | PTPN11 | SRC |  |
| AURKA | CDKN2A | FANCC | GNA13 | KMT2A (MLL) | NOTCH2 | QKI | STAG2 |  |
| AURKB | CDKN2B | FANCD2 | GNAQ | KMT2C (MLL3) | NOTCH3 | RAC1 | STAT3 |  |
| AXIN1 | CDKN2C | FANCE | GNAS | KMT2D (MLL2) | NPM1 | RAD50 | STAT4 |  |
| AXL | CEBPA | FANCF | GPR124 | KRAS | NRAS | RAD51 | STK11 |  |
| BAP1 | CHD2 | FANCG | GRIN2A | LMO1 | NSD1 | RAF1 | SUFU |  |
| BARD1 | CHD4 | FANCL | GRM3 | LRP1B | NTRK1 | RANBP2 | SYK |  |
| BCL2 | CHEK1 | FAS | GSK3B | LYN | NTRK2 | RARA | TAF1 |  |
| BCL2L1 | CHEK2 | FAT1 | H3F3A | LZTR1 | NTRK3 | RB1 | TBX3 |  |
| BCL2L2 | CIC | FBXW7 | HGF | MAGI2 | NUP93 | RBM10 | TERC |  |
| BCOR | CREBBP | FGF10 | HNF1A | MAP2K1 | PAK3 | RET | TERT  (promoter only) |  |
| BCORL1 | CRKL | FGF14 | HRAS | MAP2K2 | PALB2 | RICTOR | TET2 |  |
| BLM | CRLF2 | FGF19 | HSD3B1 | MAP2K4 | PARK2 | RNF43 | TGFBR2 |  |
| BRAF | CSF1R | FGF23 | HSP90AA1 | MAP3K1 | PAX5 | ROS1 | TNFAIP3 |  |
| BRCA1 | CTCF | FGF3 | IDH1 | MCL1 | PBRM1 | RPTOR | TNFRSF14 |  |
| BRCA2 | CTNNA1 | FGF4 | IDH2 | MDM2 | PDCD1LG2 | RUNX1 | TOP1 |  |
| BRD4 | CTNNB1 | FGF6 | IGF1R | MDM4 | PDGFRA | RUNX1T1 | TOP2A |  |
| BRIP1 | CUL3 | FGFR1 | IGF2 | MED12 | PDGFRB | SDHA | TP53 |  |
| BTG1 | CYLD | FGFR2 | IKBKE | MEF2B | PDK1 | SDHB | TSC1 |  |
| BTK | DAXX | FGFR3 | IKZF1 | MEN1 | PIK3C2B | SDHC | TSC2 |  |

<http://www.foundationone.com/genelist1.php>

**Supplemental Table 2:** 28 Gene Rearrangements detected on the Foundation Medicine Panel

| ALK | BRAF | BRD4 | ETV4 | ETV6 | KIT | MYC | NTRK2 | RARA |
| --- | --- | --- | --- | --- | --- | --- | --- | --- |
| BCL2 | BRCA1 | EGFR | ETV5 | FGFR2 | MSH2 | NOTCH2 | PDGFRA | RET |
| BCR | BRCA2 | ETV1 | ETV6 | FGFR3 | MYB | NTRK1 | RAF1 | ROS1 |

<http://www.foundationone.com/genelist1.php>
